# Molecular diagnosis of kerion celsi caused by *Trichophyton tonsurans* in a Italian child

**DOI:** 10.1016/j.mmcr.2019.04.010

**Published:** 2019-04-27

**Authors:** Laura Trovato, Salvatore Oliveri, Maria Domina, Ildebrando Patamia, Guido Scalia, Rocco De Pasquale

**Affiliations:** a U.O.C. Laboratory Analysis Unit, A.O.U. “Policlinico-Vittorio Emanuele”, Via S. Sofia 78, Catania 95123, Italy; b Department of Biomedical and Biotechnological Sciences, University of Catania, Via S. Sofia 97, Catania 95123, Italy; c U.O.C Dermatology, Vittorio Emanuele Hospital, Via Plebiscito 628, Catania 95100, Italy

**Keywords:** Tinea capitis, Kerion celsi, *Trichophyton tonsurans*, Diagnosis, Multiplex PCR

## Abstract

*T. tonsurans* is an anthropophilic dermatophyte causing several clinical variants of tinea capitis including the Kerion celsi that can be often unrecognised or confused with other lesions. We report a case of Kerion celsi caused by *Trichophyton tonsurans* in a child following an excoriation to the scalp caused by a fall in a public park. The use of multiplex PCR assay has enabled rapid diagnosis of tinea capitis from *T. tonsurans* with a result in less than 48 hours and therefore the possibility of quickly starting antifungal therapy. The patient had a complete recovery at the end of the antifungal treatment.

## 1 Introduction

Tinea capitis (TC) is an infection caused by dermatophyte fungi that affects the scalp and hair. It is typically caused by zoophilic and geophilic species of the genus *Microsporum* and *Trichophyton* and remains the most common cutaneous fungal infection in children, most prevalent between 3 and 7 years of age [1]. The epidemiology of TC mainly depends on the geographical origin of the patients. In Europe, *Microsporum canis* is the most common cause (80% of cases), although an increased number of cases caused by *Trichophyton* spp. (particularly *T. tonsurans*) have been described, possibly facilitated by immigration from geographic areas where that vector is the predominant agent of tinea capitis [[2], [3], [4]].

## 2 Case

**Fig. 1:**
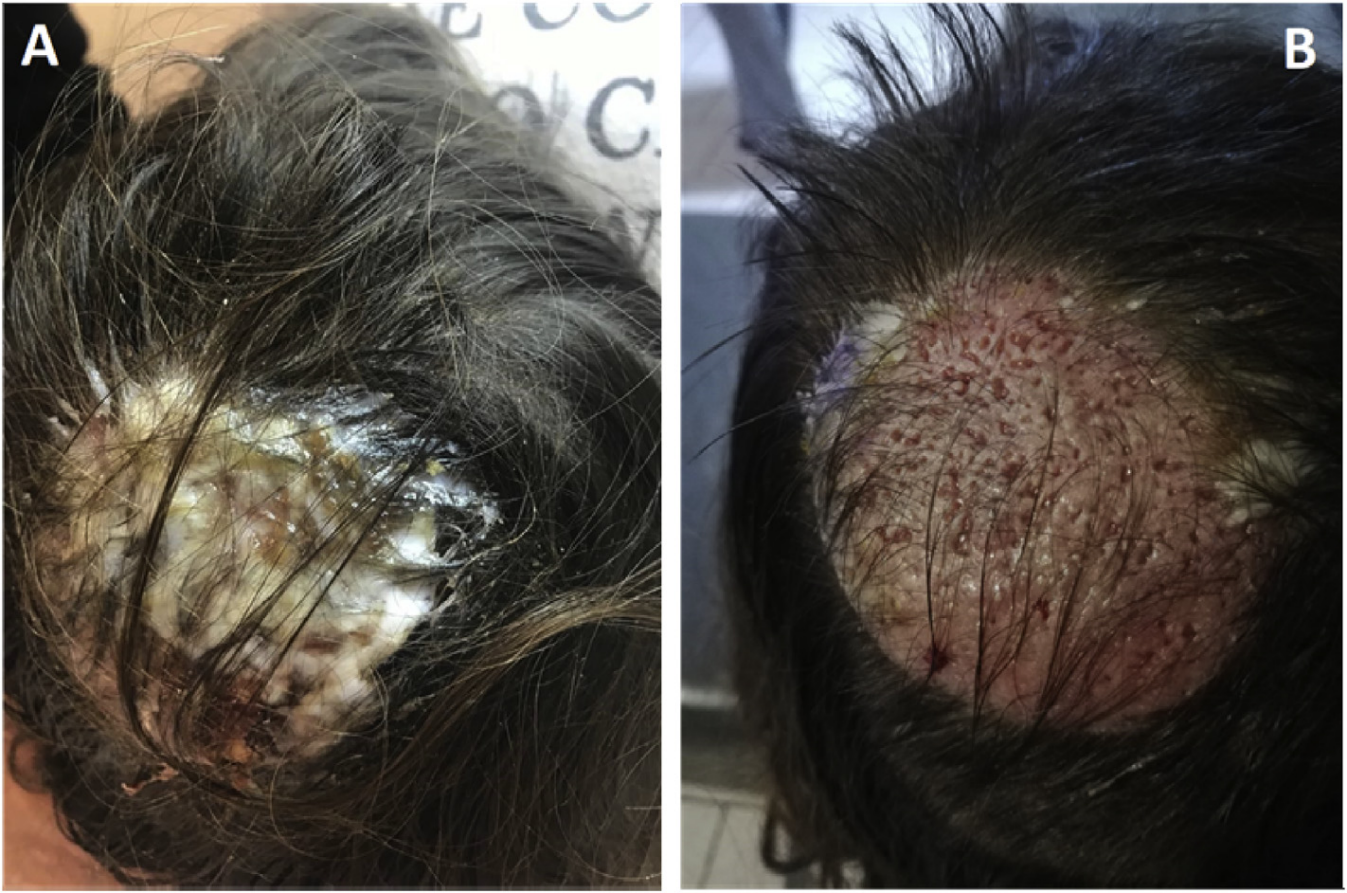
(A) The lesion on occipital area of the scalp, intensely painful on palpitation, covered with purulent secretions; (B) the lesion after cleaning with sterile normal saline (0.9% sodium chloride for injection).

A 5-year-old girl was assessed in September 2018 for the presence of an round mass (5 cm) visible in the occipital area of the scalp, with purulent secretions, and alopecia, that persisted after a week of antibiotic treatment (oral amoxicillin and clavulanic acid 50 mg/kg per day) (Fig. 1) (day 0). It was reported that the symptoms began following an excoriation caused by a fall. The patient also had fever and neutrophilic leucocytosis (white blood cells 13580 per μL [normal range 5200–12400], neutrophils 7850 per μL [normal range 1800–7700], lymphocytes 3900 per μL [normal range 1000–4800], monocytes 1300 per μL [normal range 120–1200], eosinophils 530 per μL [normal range 10–500]). A swab sample was taken from the scalp lesions for bacteriological and mycological examination. Hair was also collected (day +1). For the mycological examination conventional and molecular diagnostic methods by microscopic, fungal culture in Sabouraud's dextrose agar medium supplemented with 0.5% cycloheximide and 1% chloramphenicol and multiplex PCR assay was taken.

The DermaGenius^®^ multiplex kit (PathoNostics, The Netherlands) was use for the detection the most clinical prevalent dermatophytes species. The DNA was extracted by using the PathoNostics Extraction Kit following the manufacturer's instructions. The multiplex PCR was performed according to manufacturer's instructions: 5 μl of DNA extract was added to the PCR mix and a Rotor-Gene Q (Qiagen) was used for amplification and melting curve analysis. A positive control and negative template control (NTC) were included in each PCR run.

The direct microscopic examination with 15% potassium hydroxide and glycerol showed few hyaline septate hyphae (day +1). Also the examination of lesional hair showed a positive endothrix result. The bacterial culture of the buffer was negative while *Trichophyton tonsurans* was detected by multiplex PCR (day +2).

**Fig. 2:**
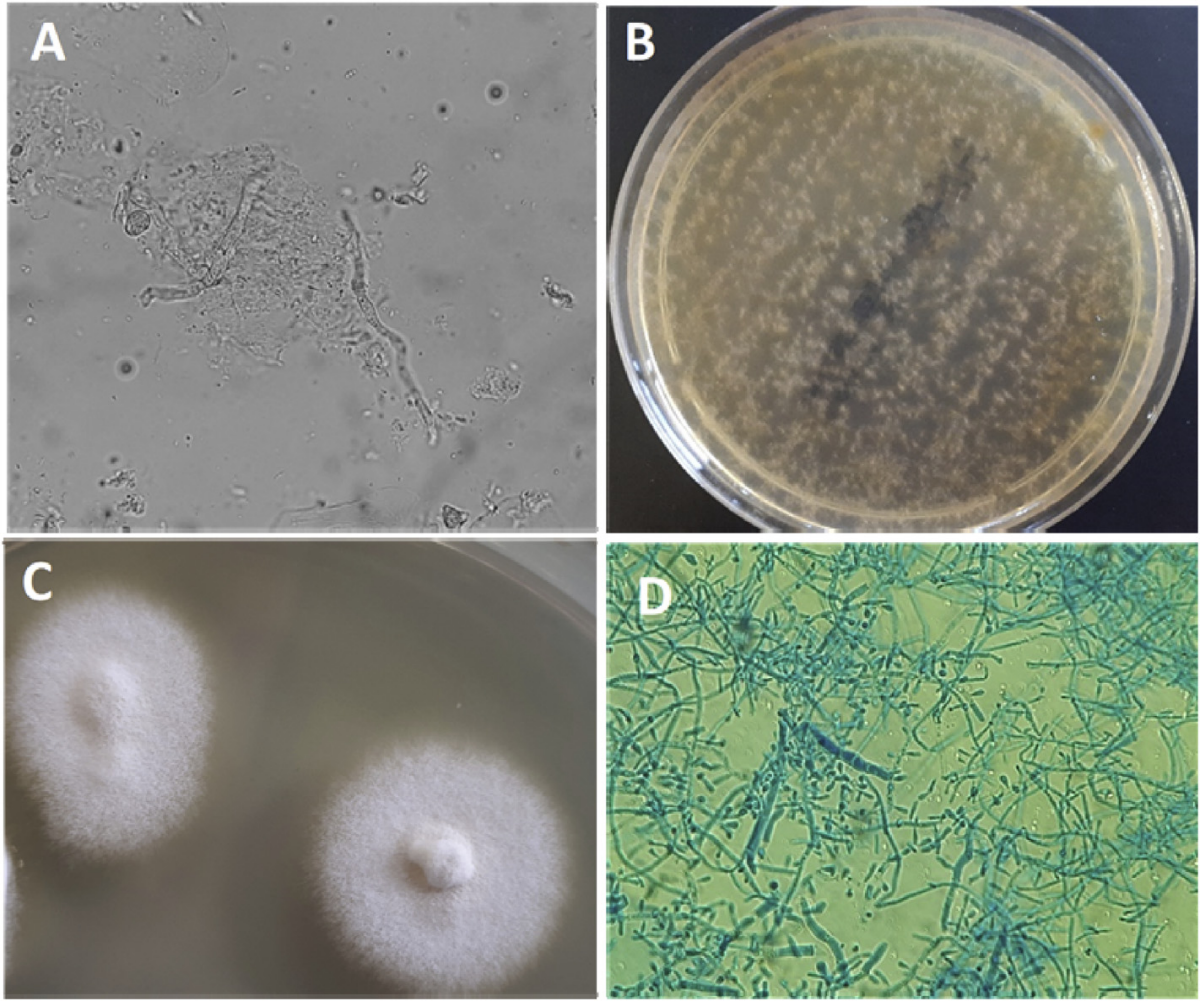
Direct examination of skin scalp swab with 15% KOH and glycerol (A) (original magnification ×40), (B) growth on Sabouraud Dextrose Agar with cycloheximide and chloramphenicol after 5 days of incubation at 32 °C; colonial morphology of a subculture of *T. tonsurans* at 10 day (C); microscopic structure of the colony showing numerous microconidia and few macroconidia of *T. tonsurans*.

After 5 day of incubation at 30 °C the colonies started to grow on Sabouraud's dextrose agar supplemented with cycloheximide and chloramphenicol, initially appearing as a grey, flat and powdery colony without red pigment (day +6). Identification of the isolate was performed by standard phenotypic methods, based on the macroscopic and microscopic morphological study. In particular, the microscopic morphological study showed that microconidia were produced in abundance, most forming loosely clustered branches, sessile, clavate, cylindrical. Few clavate macroconidia, thin-walled, was observed (day +16). The pathogen was identified as *T. tonsurans*. The results of mycological examinations are shown in Fig. 2.

**Fig. 3:**
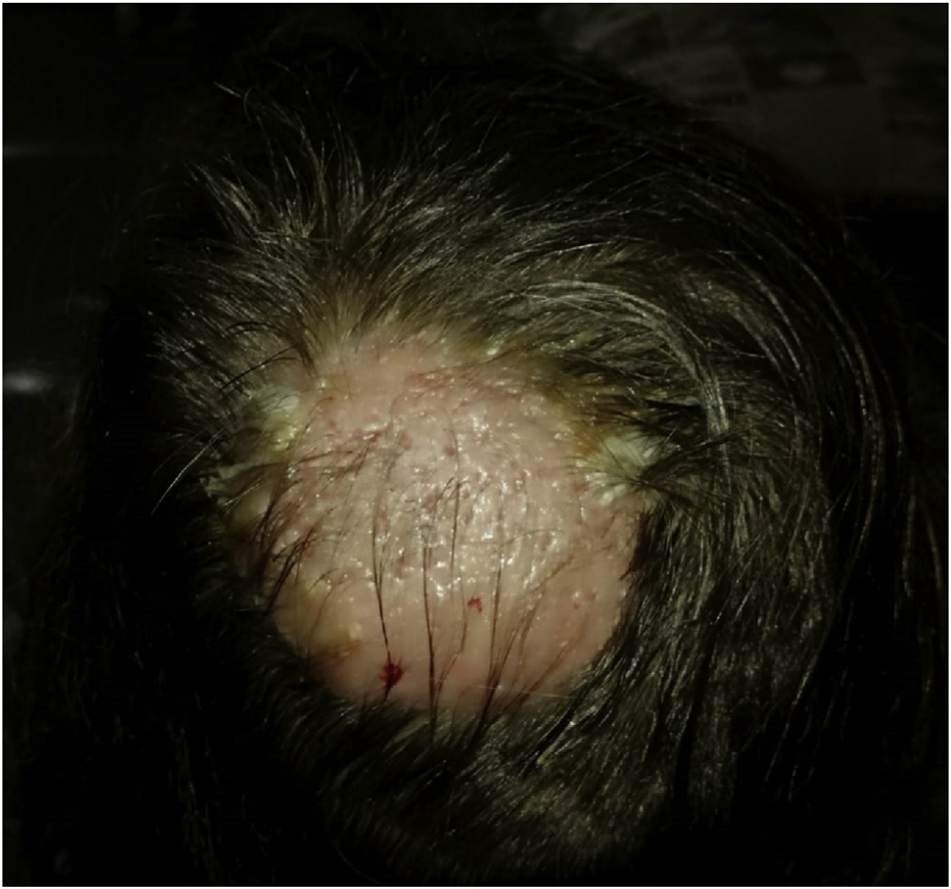
Clinical improvement of the lesion after 2-week treatment with Griseofulvin.

Matrix-assisted laser desorption ionization time of flight mass spectrometry (MALDI-TOT MS) on a Microflex LT (Bruker Daltonics, Bremen, Germany) platform after ethanol-formic acid extraction identified the isolate as *Trichophyton tonsurans* (score: 2.10) (day +6). A significant clinical improvement was observed after a 8-week course of systemic griseofulvin (oral, 10 mg/die) and a 6-week course of topical econazole (1% cream, three times a day) (Fig. 3).

## 3 Discussion

Tinea capitis is the most important superficial fungal condition in children. Although *Microsporum canis* is the most common encountered causative agent in TC in Europe (80%), an increased number of cases caused by anthropophilic dermatophytes (particularly *T. tonsurans*) have been described. *T. tonsurans* is an anthropophilic dermatophyte causing several clinical variants of tinea capitis including the Kerion celsi. Despite this increased number of case of tinea capitis by *T. tonsurans* in Europe have been described, few case reports about the this anthropophilic dermatophyte have been reported in Italy [5,6].

We report a case of Kerion celsi caused by *Trichophyton tonsurans* in a child following an excoriation to the scalp caused by a fall in a public park. Kerion celsi is often unrecognised or confused with other lesions and this can lead to underdiagnosis with subsequently delayed or inappropriate treatment (as happened with our patient who was treated with antibiotic without improvement). Generally, the diagnosis of tinea capitis must be confirmed by KOH preparation of scalp and infected hairs and a fungal culture. Cultures, which are widely used in clinical laboratories as the reference test, remain negative in 20–30% of positive microscopy cases, as well as being associated with a long turnaround time (TAT). In our case, the use of multiplex PCR assay confirmed the tinea capitis from *T. tonsurans* with a result in less than 48 hours and therefore the possibility of quickly starting antifungal therapy. The clinical evaluation of use of the DermaGenius^®^ multiplex kit has been performed only in nail samples [7]. Our case shows that multiplex PCR it can be also be used on skin and hair samples when a clinician needs a fast and precise diagnostic and when a patient is already under antifungal treatment.

The transmission of *T. tonsurans* may occur directly (by infected or asymptomatic human carriers) or indirectly (fomites). We questioned the mother of the child to determine the possible origin of his infection. A possible source of the infection we thought was that contact of his scalp with the soil due to the fall. In fact, *T. tonsurans* easily survives outside the host and it is reported the only anthropophilic fungus isolated in soil samples from public sites, together with geophilic or zoophilic fungi [8]. Then, as was done for other fungi [9], it might be useful to evaluate *T. tonsurans* presence in the environment to understanding the ecology of this fungal pathogen, its ecological niches and therefore the source of the infection although this is an anthropophilic fungi. Another hypothesis is that the child was an asymptomatic carrier of *T. tonsurans* in the scalp and that the excoriation following the fall triggered the infection. The standard treatment for kerion celsi is griseofulvin (20–25 mg/kg per day of microsize tablets or 10–15 mg/kg per day of ultramicrosize tablets for 6–8 weeks). Our patient had a prominent clinical improvement to this regime with clinical and mycological cure and used an antifungal shampoo until lesion resolved.

## Conflict of interest

There are none.
